# Inosine Triphosphate Pyrophosphohydrolase (*ITPA*) polymorphic sequence variants in adult hematological malignancy patients and possible association with mitochondrial DNA defects

**DOI:** 10.1186/1756-8722-6-24

**Published:** 2013-03-29

**Authors:** Mazin A Zamzami, John A Duley, Gareth R Price, Deon J Venter, John W Yarham, Robert W Taylor, Laurence P Catley, Timothy HJ Florin, Anthony M Marinaki, Francis Bowling

**Affiliations:** 1 King Abdulaziz University, Jeddah, Saudi Arabia; 2 The University of Queensland, Brisbane, Australia; 3 Mater Medical Research Institute, Brisbane, Australia; 4 Pathology, Mater Health Services, Brisbane 4101, Australia; 5 Wellcome Trust Centre for Mitochondrial Research, Institute for Ageing and Health, Newcastle University, Newcastle Upon Tyne, UK; 6 Purine Research Laboratory, GSTS Pathology, Guy’s and St Thomas’ Hospitals, HeLaLondon, UK

**Keywords:** ITPA, Mitochondria, Haematological malignancy, Microarray, N-call

## Abstract

**Background:**

Inosine triphosphate pyrophosphohydrolase (ITPase) is a ‘house-cleaning’ enzyme that degrades non-canonical (‘rogue’) nucleotides. Complete deficiency is fatal in knockout mice, but a mutant polymorphism resulting in low enzyme activity with an accumulation of ITP and other non-canonical nucleotides, appears benign in humans. We hypothesised that reduced ITPase activity may cause acquired mitochondrial DNA (mtDNA) defects. Furthermore, we investigated whether accumulating mtDNA defects may then be a risk factor for cell transformation, in adult haematological malignancy (AHM).

**Methods:**

DNA was extracted from peripheral blood and bone marrow samples. Microarray-based sequencing of mtDNA was performed on 13 AHM patients confirmed as carrying the *ITPA 94C>A* mutation causing low ITPase activity, and 4 AHM patients with wildtype *ITPA*. The frequencies of *ITPA 94C>A* and *IVS2+21A>C* polymorphisms were studied from 85 available AHM patients.

**Results:**

*ITPA 94C>A* was associated with a significant increase in total heteroplasmic/homoplasmic mtDNA mutations (p<0.009) compared with wildtype *ITPA*, following exclusion of haplogroup variants. This suggested that low ITPase activity may induce mitochondrial abnormalities. Compared to the normal population, frequencies for the *94C>A* and *IVS2+21A>C* mutant alleles among the AHM patients were higher for myelodyplastic syndrome (MDS) - but below significance; were approximately equivalent for chronic lymphoblastic leukemia; and were lower for acute myeloid leukemia.

**Conclusions:**

This study invokes a new paradigm for the evolution of MDS, where nucleotide imbalances produced by defects in ‘house-cleaning’ genes may induce mitochondrial dysfunction, compromising cell integrity. It supports recent studies which point towards an important role for ITPase in cellular surveillance of rogue nucleotides.

## Introduction

**Figure 1:**
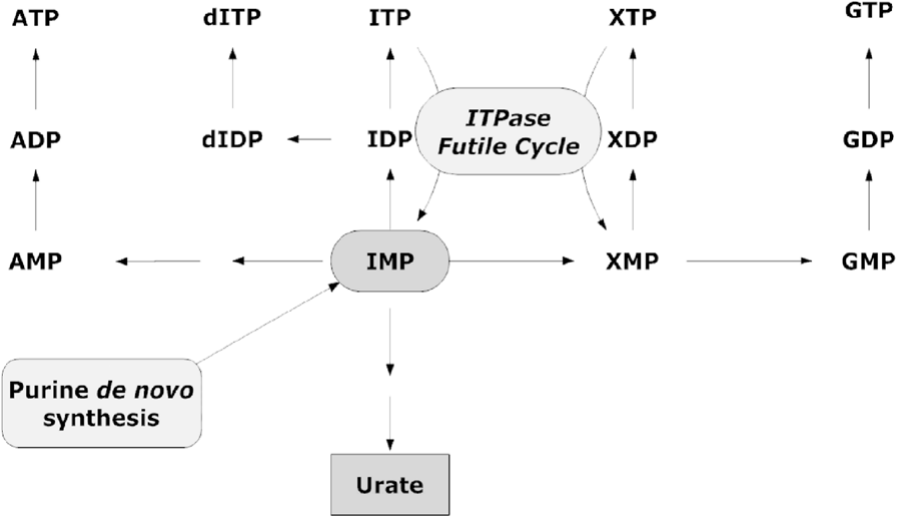
**Overview of purine metabolism and ITPase ‘futile cycle’.** ITPase: Inosine triphosphate pyrophosphohydrolase; IMP: Inosine monophosphate; XMP: Xanthosine monophosphate; GMP: Guanosine monophosphate; dITP: deoxy-Inosine triphosphate; AMP: Adenosine monophosphate.

During purine nucleotide synthesis in cells, the non-canonical nucleotide inosine triphosphate (ITP) can be synthesized from inosine monophosphate (IMP) by the sequential actions of monophosphate kinase and nucleoside diphosphate kinase. ITP pyrophosphohydrolase (ITPase) catalyses the conversion of ITP back to IMP forming a ‘futile cycle’ (Figure 1) [1]. Importantly, ITPase also acts as a house-cleaning enzyme by degrading other ‘rogue’ purine nucleotides in cells, e.g., endogenous deoxy-inosine triphosphate (dITP) and deoxy-xanthosine triphosphate (dXTP) [2-4]. In addition, exogenous nucleotides derived from purine drugs, such as thio-ITP and methyl-thio-ITP derived from thiopurines, are substrates for ITPase [5]. More recently it has been shown that low ITPase appears to protect against ribavirin-induced hemolytic anaemia, possibly through a mechanism that binds ribavirin as its rogue nucleotide form [6].

Knockout studies of ITPase in the mouse have shown that deficient mice show severe growth retardation and die before weaning [7], which was attributed to accumulation of mutagenic dITP in cellular deoxy-nucleotide pools [8]. But despite a critical role in the surveillance of rogue nucleotides, genetically low ITPase activity in humans is relatively common and has been assumed to be clinically benign, presenting only as an autosomal recessive red cell enzymopathy. Abnormal accumulation of ITP in humans who have low ITPase activity has been shown to occur in both erythrocytes and leucocytes, implying that inherited low ITPase occurs in all tissues [9]. Interestingly, *ITPA* has been mentioned in relation to hematological disease where it has been noted as one of five mixed-lineage leukemia associated genes whose up-regulation accompanies amplification of the *MLL* gene region of 11q23 [10]. Further evidence has been provided by a recent *in vitro* study on human HeLa cells, with a knockdown *ITPA* gene, which showed that the absence of functional ITPase activity can lead to accumulation of non-canonical nucleotides which may cause DNA damage and cancer [11].

Five single polymorphic sequence variants in the human *ITPA* gene have been identified, two of which are associated with ITPase loss of activity (*94C>A* in exon-2, and *IVS2+21A>C*). These interact and affect branch points resulting in missplicing of exons 2 and 3 leading to shortening of polypeptide stretches in the enzyme [12]. The other three coding region *ITPA* polymorphic sequence variants are silent mutations (*138G>A, 561G>A and 708G>A*)[3]. Homozygosity of the *ITPA 94A* allele, which results in deficiency of ITPase activity in erythrocytes and lymphocytes, occurs in approximately 1 in 1000 Caucasians [3]. Carriers represent about 1 in 15 (6.0%) of Caucasian populations, and have an average red cell ITPase activity of about 22% of normal. This allele is more common in Asian populations, with a frequency of 11-15% [13]. Homozygosity of the *ITPA IVS2+21C* allele occurs in approximately 1 in 250 Caucasians, with a carrier frequency of about 1 in 8 (13.0%), resulting in partial reduction of ITPase activity with an average of 60% normal levels in red cells. Its frequency varies greatly in other populations, e.g. the SNP is absent among Japanese. Compound heterozygotes (*ITPA* 94A/*IVS2+21C)* have 10% of normal activity.

Mitochondria are synthesized from both nuclear genes that encode mitochondrial proteins and from a small number of genes encoded by the mitochondrial genome (mtDNA) [14]. The high mutation rate associated with mtDNA is thought to be the result of inappropriate incorporation of rogue nucleotides into the mtDNA [15] which may be caused by: (a) exposure to high concentrations of reactive oxygen species (ROS) generated in the mitochondria from the electron transfer system [16], or (b) lack of histone protection of the mitochondrial genome [17]. Repair mechanisms for mtDNA were thought to be limited [18-20], but mitochondria appear to have mechanisms that respond to mtDNA damage - rather than to genotoxic stress by itself - and re-localize repair proteins from the cytosol to the organelle [21]. These include imported mechanisms for base excision repair, now considered to include long-patch as well as short-patch, and mismatch repair [22].

Mitochondria with differing genetic backgrounds (heteroplasmy) can coexist within a cell or tissue [20], but during cell division the proportions of differing mitochondria segregating into new cells may change, resulting in some cases with daughter cells receiving only one type of mitochondria, i.e., homoplasmy [18]. Where harmful mtDNA mutations occur, segregation of the ‘mutant’ mitochondria may result in their accumulation in a significant percentage of the cells in a tissue, which may result in sufficient dysfunction to cause disease, once a critical threshold is reached [23]. For example, severe nucleotide imbalances in mitochondria can result in rapid accumulation of mutations and deletions which lead to mitochondrial failure and cell apoptosis. Progression of the mitochondriopathy can lead to multi-organ involvement. This is exemplified by mitochondrial neurogastrointestinal encephalomyopathy (MNGIE) [24], which arises from a nucleotide imbalance caused by mutation of nDNA-encoded enzyme thymidine phosphorylase (*TP*) that is imported into mitochondria [25].

No direct cause-effect relationship has been established between mitochondrial mutation and haematological malignancy. Nonetheless, progression of haematological malignancy is characterised by mtDNA mutations with a low heteroplasmic mutation load in early stages of myelodysplastic disease (MDS); however advanced stages of MDS are accompanied by a dramatic increase in the mtDNA mutations, reaching homoplasmy with acute myeloid leukemia (AML) [26].

Mutations in genes which produce metabolic imbalances or unusual types of nucleotides are thought to represent risk factors for the development of cancers, particularly where chromosome rearrangement or gene instability is implicated [27,28]. This is supported by a recent study of ITPase deficient mouse embryonic fibroblasts, which has linked rogue nucleotides produced by reduced ITPase activity to an increase of chromosomal abnormality frequency and accumulation of single-strand breaks in nuclear DNA (nDNA) [8].

We hypothesised that because of its role in rogue nucleotide surveillance, reduced ITPase activity may be detrimental to tissue mitochondrial health by accumulation of mtDNA defects, from chronic exposure to endogenous rogue nucleotides (ITP/dITP/XTP). We focused on defects found in mitochondria because surveillance and repair of mtDNA is relatively lacking compared to genomic DNA.

Furthermore, we hypothesized that the effect would be cumulative with time, i.e. more prevalent in adults. We also considered whether compromise of mitochondrial function may then be a risk factor for cell transformation. Several types of adult haematological malignancy (AHM) are already known to be related to nDNA mutations. Thus, to study mtDNA changes we focused on chronic lymphocytic leukemia (CLL), AML and MDS because of their relative frequency and association with DNA repair pathologies [29,30].

## Methods and materials

### Patient samples

Table 1 shows the patient characteristics for the samples used. In this pilot study, all suitable patient samples belonging to 3 disease groups, MDS (n=39), CLL (n=28) and AML (n=18), as available in Brisbane, were obtained from the Australasian Leukaemia and Lymphoma Group Tissue Bank, Princess Alexandra Hospital, and the Hematology Clinic at Mater Adult Hospital. DNA was extracted from a total of 85 peripheral blood or bone marrow samples. Patients were selected on the basis of: (a) Caucasian - to minimize differences in rates of ITPA sequence variants known to occur between some races [13]; (b) untreated or not participating in any trials, i.e. the disease was not secondary to prior drug therapy for malignancy; (c) age-onset haematological disease. The study was approved by the Tissue Bank Human Ethics Review Committee (HREC) and by the Mater Hospital and University of Queensland HRECS.

**Table 1:** Characteristics of a cohort of 85 adult hematological malignancy patients participating in this study

**Attributes**	**MDS**	**CLL**	**AML**
Number	39	28	18
Age (year):	75 (54–90)	68 (37–84)	69 (39–85)
median (range)			
Sex: (Male/Female)	22/17	21/7	7/11
Sample type:			
Peripheral blood	30	23	17
Bone marrow	9	5	1
Classification (n)	RAEB (n=3), RAEB-1 (n=3),	CLL (n=28)	AMLAMDS (n=9),
	RAEB-2 (n=7), MDP5q (n=1),		AMLDN (n=6),
	RCMD (n=13), MDSU (n=2),		AML16 (n=1),
			AML/TLD (n=2)
	RA (n=1), RARS(n=2),		
	CMML (n=6),CMML-1 (n=1)		

MDS: Myelodysplastic syndromes; RA: Refractory anaemia; RARS: Refractory anaemia with ringed sideroblasts; RAEB: Refractory anaemia with excess blasts; RCMD: Refractory cytopenia with multilineage dysplasia; MDSU: Myelodysplastic syndrome unclassifiable; MDP5q: Myelodysplastic syndrome associated with isolated (del)5q chromosomal abnormality; CMML: Chronic myelomonocytic leukaemia; AML: Acute myeloid leukaemia; AML/TLD: Acute myeloid leukaemia with trilineage dysplasia; AMLAMDS: Acute myeloid leukaemia with multilineage dysplasia with prior myelodysplasia; AMLDN: Acute myeloid leukaemia with multilineage dysplasia without prior myelodysplasia; AML16: AML with abnormal bone marrow eosinophils (inv)16(p13q22) or t(16;16)(p13;q22); (CBFb/MYH11); CLL: Chronic lymphocytic leukaemia.

### DNA extraction and ITPA (PCR) amplification

DNA was extracted using a QIAamp DNA Blood Mini kit (Qiagen, Valencia, CA). The *ITPA 94C>A* and *IVS2+21A>C* sequence variants were screened by standard PCR using primers to amplify exons 2, 3 and 4 and the intervening sequences (introns), by a method modified from Sumi *et al.,*[3]. Identification of sequence variants was performed by sequencing of the amplified regions, using BigDye® terminator v3.1 cycle sequencing, as per manufacturer’s instructions then analysed with Chromas v2.31 and ChromasPro v1.42 software.

### Complete mtDNA (PCR) amplification and purification and affymetrix microarray protocol

The entire mitochondrial genome was analysed from the 13 AHM patients confirmed as carrying the *ITPA 94A* mutant allele and from 4 randomly-selected AHM patients with the *ITPA 94C* wildtype allele. Analysis of mtDNA mutations was achieved using the Affymetrix MitoChip v2.0 resequencing array, following the manufacturer’s protocol [31,32]. Preparation of the mtDNA involved amplifying the entire mtDNA sequence in three overlapping long PCR fragments using TaKaRa LA *Taq* (TaKaRa Biomedicals), with each reaction containing 25ng of genomic DNA. The three primers sets are described in the Affymetrix MitoChip v2.0 supporting documentation. After target amplification, PCR products were purified using a High Pure PCR Product Purification Kit (Roche, Germany). The hybridization steps of the Affymetrix protocol can be summarized as follows: PCR products were pooled, to ensure equal molar amount, followed by fragmentation and labeling of the pooled PCR products, then hybridization for 16 hours at 49°C with 60 rpm rotation in a hybridization mix solution (fragmentation and labeling enzymes and reagents were part of the Affymetrix GeneChip Resequencing Reagent Kit). Washing of the MitoChip was performed using the Affymetrix Fluidics Station 450 (Mini_DNAArray_WS5_450), and finally, scanning the MitoChip array for the four alternative oligonucleotides for each single base using Affymetrix GeneChip Scanner 3000.

### Automated batch analysis of microarray data

Data analysis of the microarrays was carried out using Affymetrix GSEQ software. The Affymetrix MitoChip v.2.0 is tiled with 25-mer DNA probes divided into two sections, the first section being the Cambridge Reference Sequence (rCRS) NC_012920 for mtDNA, while the second section comprises sequences representing 500 of the most common haplotypes recorded in the MitoMap public database (http://www.mitomap.org). These include known mtDNA single nucleotide polymorphisms (SNPs) as well as known pathological mutations, insertions, and deletions. The Affymetrix algorithm parameter settings recommended to achieve optimal performance were used to analyze the mitochondrial sequences, with ‘diploid’ selected as the genome model to enable the detection of heteroplasmy. Using IUPAC codes, the GSEQ MitoChip v2.0 microarray software will assign: homoplasmic mutations with normal bases (A, C, G or T); heteroplasmic mutations, signified by IUPAC codes R (A/G), Y (C/T/U), K (G/T/U), M (A/C), S (C/G), or W (A/T/U); or an N-Call where a base position cannot be assigned by the software (the letter “N” in the sequence indicating signal intensity within two standard deviations of zero “no signal”). A Quality Score Threshold (QST) of 3 provided the highest performance in terms of overall base calling accuracy and call rates by the software [19].

### Haplogroup analysis

The mtDNA from AHM patient samples were subjected to haplogroup analysis to determine which of the observed mutations could be considered genetically unique for each patient, by removing those known to be associated with the patient’s haplogroup (and can therefore be considered non-pathogenic). This analysis was performed using the freely available HaploGrep web application (http://haplogrep.uibk.ac.at/) [33], which is based on data from Phylotree (http://www.phylotree.org/) [34]. The remaining unique pathogenic and non-pathogenic mtDNA mutations were then considered according to the hypothesis that the *ITPA* mutation causes an increase in mutagenesis (see Additional file 1: Tables S1-S3). Non-parametric analysis (Mann Whitney two-tailed test) was then performed for comparing the statistical significance of differences in mtDNA mutation rates.

### sPROFILER software for N calls analysis

Data from GSEQ base calls was further analysed using sPROFILER (strand-specific PRObe cell intensity comparison for FILtERing). This is a novel algorithm developed by Kothiyal *et al.*[35] for improving array call rates using MATLAB, a numerical computing and programming language, over GSEQ call rates, which is based on intensity signature. When a base cannot be called because of poor hybridization on one of the strands, a threshold is determined by using the next highest intensity ratio on either strand to determine the base call. sPROFILER helped to resolve >80% of N-calls from GSEQ and allowed 99.6% of sequence to be assigned. sPROFILER was designed to not query base calls conforming to the reference sequence (rCRS), as GSEQ is conservative in assigning a base call.

### Sample size

Sample size was based on detecting the *ITPA 94C>A* and *IVS2+21A>C* polymorphisms in each individual hematological malignancy group using a 1-sided Fishers Exact Test. We previously determined the frequencies of *ITPA 94C>A* and *IVS2+21A>C* polymorphisms in a normal Caucasian population [3]. For the present study, it was calculated that a sample size of n= 100 alleles (i.e. 50 patients) in each disease group would provide 80% power (p=0.05) to distinguish a raised minimum frequency of 15% for the *94C>A* SNP and 25% for *IVS2+21A>C*. It was possible to attain sample numbers approaching 50 for the MDS group (n=39, or 78 alleles), but CLL (n=28, 56 alleles) and AML (n=18, 36 alleles) sample numbers were restricted, thus requiring higher frequencies of the *ITPA* sequence variants to attain significance.

## Results

### Effect of ITPA polymorphic sequence variants on mitochondrial DNA mutation rates

The study subjects’ anthropometric, types and characteristics are summarized in Table 1 and Table 2. To examine the effect of reduced ITPase activity on mtDNA, the 13 confirmed AHM patients with the *ITPA 94A* mutant allele, comprising 11 heterozygotes and 2 compound heterozygotes, were compared with 4 AHM patients homozygous for the *ITPA 94C* wildtype allele (and IVS2+21A wildtype allele) (Table 2). Peripheral blood or bone marrow samples were excluded from examination of mtDNA mutation rates if the intronic *ITPA* variant (*IVS2+21A>C*) was present, because both homozygotes and carriers exhibit only partial reduced ITPase activity (Table 1).

**Table 2:** Adult hematological malignancy patient characteristics of 17 cell samples selected for study

**Attributes**	**MDS**	**CLL**	**AML**	**Control**
Number	8	4	1	4
Age (year):	75 (54–90)	68 (37–84)	69 (39–85)	65 (54–79)
median (range)				
Sex (Male/Female)	3/5	3/1	1/0	3/1
Sample type:				
Peripheral blood	7	3	1	2
Bone marrow	1	1	0	2
Classification (n)	RAEB-1 (n=1)	CLL (n=4)	AMLDN (n=1)	MDS (n=2, RCMD & RAEB-2)
	RAEB-2 (n=2)			
	MDP5q (n=1),			AML (n=1, AML/TDL)
				CLL (n=1)
	RCMD (n=2),			
	MDSU (n=1)			
	CMML (n=1)			

Peripheral white cells were randomly selected from 13 patients carrying the *ITPA 94A* variant allele and 4 ‘control’ patients homozygous for the *ITPA 94C* wildtype allele. MDS: Myelodysplastic syndromes; RAEB: Refractory anaemia with excess blasts; RCMD: Refractory cytopenia with multilineage dysplasia; MDP5q: Myelodysplastic syndrome associated with isolated (del)5q chromosomal abnormality; MDSU: Myelodysplastic syndrome unclassifiable; CMML: Chronic myelomonocytic leukaemia; AML: Acute myeloid leukaemia; AML/TLD: Acute myeloid leukaemia with trilineage dysplasia; AMLDN: Acute myeloid leukaemia with multilineage dysplasia without prior myelodysplasia; CLL: Chronic lymphocytic leukaemia.

**Figure 2:**
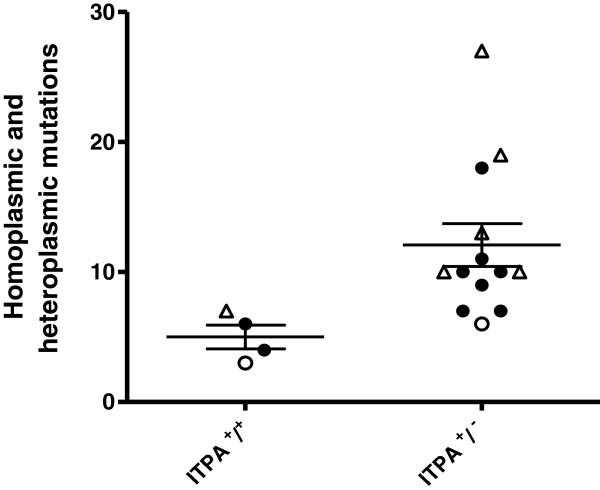
**Total homoplasmic and heteroplasmic mutations in mtDNA of AHM patients.** 13 AHM patients carrying the *ITPA 94A* variant allele (i.e. *ITPA+/−*) comprised MDS (closed circles) n=8, CLL (open triangles) n=4, AML (open circles) n=1. These were compared with 4 patients homozygous for the *ITPA 94C* wildtype allele (i.e. *ITPA+/+*), comprising MDS n=2, CLL n=1 and AML n=1. Mutations were identified against the MitoChip standard mtDNA reference sequence (rCRS) and those belonging to the assigned mtDNA haplogroup of each patient excluded. The results are shown as means ± SEM (p < 0.009, Mann–Whitney two-tailed test).

**Figure 3:**
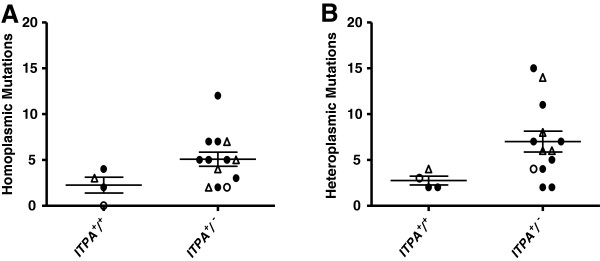
**Homoplasmic and heteroplasmic mutations in mtDNA of AHM patients.** This shows separate analyses of (**A**) homoplasmic and (**B**) heteroplasmic mutations in mtDNA of 13 AHM patients carrying the *ITPA 94A* variant allele, comprising MDS (closed circles) n=8, CLL (open triangles) n=4, and AML (open circles) n=1, compared with 4 patients homozygous for the *ITPA 94C* wildtype allele, comprising MDS n=2, CLL n=1 and AML n=1. Mutations were identified against the MitoChip standard mtDNA reference sequence (rCRS) and those belonging to the assigned mtDNA haplogroup of each patient excluded. The results are shown as means ± SEM (p = 0.068 for homoplasmic mutations, not significant; p < 0.035 for heteroplasmic mutations, Mann–Whitney two-tailed test).

Following exclusion of sequence variants signifying assigned haplogroups, the number of both heteroplasmic and homoplasmic mutations were plotted (Figure 2) and compared using a Mann–Whitney two-tailed test. This showed that there were statistically higher numbers of mtDNA mutations in the patients carrying the *ITPA* mutant allele (P < 0.009) than in the patients carrying the *ITPA* wild-type allele. For 10 of the 13 patients carrying the *ITPA 94A* allele compared with the 4 patients lacking this allele, there was a significant increase in heteroplasmic mtDNA mutations (p < 0.035), as well as an increase in the number of homoplasmic mutations but this increase was not significant (p = 0.068) (Figure 3).

In summary for the 13 AHM patients carrying the *ITPA 94A* mutant allele, a total of 149 mtDNA nucleotide changes were detected, including 84 mutations previously undescribed in the website (http://www.mitomap.org). This compared with 4 patients homozygous for the *ITPA 94C* wildtype allele, where a total of 20 mtDNA nucleotide changes were found, including 12 undescribed mutations and 7 insertions. Interestingly, the number of mutations in the tRNA and rRNA encoding regions of the mtDNA of ITPA deficient patients had increased from zero to a total of 20 mutations in heteroplasmic and 11 mutations in homoplasmic mitochondria.

[All the mtDNA changes that were identified for all AHM patients are available in online Additional file 1: Tables S1-S3.]

**Figure 4:**
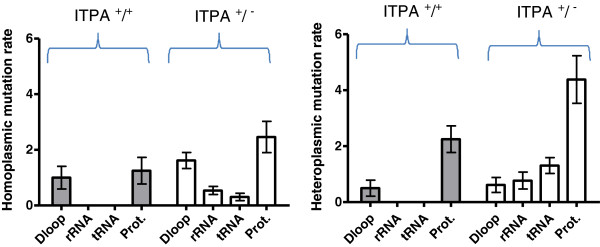
**Rates of homoplasmic and heteroplasmic mutations per AHM patient affecting specific mtDNA regions.** (Left) Homoplasmic and (Right) heteroplasmic mutation rates in specific mtDNA regions, for 13 AHM patients carrying the *ITPA 94A* mutant allele (MDS n=8, CLL n=4 and AML n=1) compared with 4 AHM patients homozygous for the *ITPA 94C* wildtype allele (MDS n=2, CLL n=1 and AML n=1). D-loop: control region; rRNA: mitochondrial ribosomal RNAs; tRNA: mitochondrial transfer RNAs; Prot: protein gene. Mutations were identified against the MitoChip standard mtDNA reference sequence (rCRS) and those belonging to the assigned mtDNA haplogroup of each patient excluded. The results are shown as means ± SEM (p values not significant, Mann–Whitney two-tailed test).

Figure 4 shows the mtDNA regions of homoplasmic and heteroplasmic changes (control region, mitochondrial ribosomal RNAs, mitochondrial transfer RNAs and protein genes) comparing AHM patients carrying the *ITPA 94A* allele versus the *94C* allele. These nucleotide changes can be calculated as mutational ‘rates’ (number of base changes per patient). Mutation rates in patients carrying the *ITPA 94A* allele appeared higher, particularly in 3 of 4 mtDNA regions (rRNA, tRNA, Prot), but these differences were not significantly compared to wildtype *ITPA* patients.

However, analysis of the frequency of nucleotide changes among the AHM patients showed they were spread unevenly in the entire mtDNA, with a frequency (% mutations per gene) varying from 0.02 - 1.6% (Table 3). In particular, the MitoChip v2.0 analysis revealed mtDNA “hotspot regions” in the distribution of mutations when the *ITPA 94C>A* sequence variant was present. These hotspots occurred as raised mean mutation rates in protein encoded genes *MT-ND4, MT-ND5,MT-ND6*, *MT-CO1*, *MT-CO2, MT-CO3,MT-ATP6* and *MT-CYB*, that may compromise their activity.

**Table 3:** Mutation rates in mtDNA genes of AHM patients

**mtDNA genes**		**Patients carrying**			**Patients homozygous for**		
	**Gene length**	***ITPA 94A *variant allele: mtDNA mutations**			***ITPA 94C *wildtype allele: mtDNA mutations**		
(rCRS)	(bp)	Mean number	SEM	Frequency	Mean number	SEM	Frequency
D-Loop	*1098*	2.23	0.48	0.20	1.50	0.50	0.14
MT-RNR1	*954*	0.38	0.24	0.04	0.00	0.00	0.00
MT-RNR2	*1558*	0.85	0.25	0.05	0.00	0.00	0.00
MT-ND1	*956*	0.15	0.10	0.02	0.25	0.25	0.03
MT-ND2	*1042*	0.46	0.22	0.04	1.50	0.29	0.14
MT-CO1	*1542*	0.23	0.17	0.01	0.00	0.00	0.00
MT-CO2	*684*	0.23	0.12	0.03	0.00	0.00	0.00
MT-ATP6	*680*	0.54	0.22	0.08	0.25	0.25	0.04
MT-CO3	*784*	0.31	0.17	0.04	0.25	0.25	0.03
MT-TR	*65*	0.23	0.17	0.35	0.00	0.00	0.00
MT-ND4L	*290*	0.08	0.08	0.03	0.00	0.00	0.00
MT-ND4	*1378*	1.00	0.34	0.07	0.00	0.00	0.00
MT-TS2	*59*	0.08	0.08	0.13	0.00	0.00	0.00
MT-TL2	*71*	1.15	0.27	1.63	0.00	0.00	0.00
MT-ND5	*1812*	1.77	0.34	0.10	1.00	0.41	0.06
MT-ND6	*525*	0.46	0.22	0.09	0.00	0.00	0.00
MT-CYB	*1141*	1.46	0.43	0.13	0.25	0.25	0.02
MT-TT	*66*	0.15	0.15	0.23	0.00	0.00	0.00
Total	*14705*	11.77*	4.05	0.08	5.00	2.20	0.03

The distribution of mutations in mtDNA genes is shown in 13 patients carrying the *ITPA 94A* variant allele compared with 4 patients homozygous for the *ITPA 94C* wildtype allele. Results shown as number of mutations per gene (mean and SEM) and frequency (% mutations/base). *p < 0.004, using Two-way ANOVA. Abbreviations of mitochondrial genes according to international notation (http://www.mitomap.org*)*.

When nucleotide changes in the mtDNA sequences from both sets of AHM patients were compared for the reference sequence (Table 3), the mean number of mtDNA aberrations in AHM patients carrying the aberrant *ITPA 94A* allele (mean = 11.8) was significantly higher than the patients homozygous for the wildtype *ITPA 94C* allele (mean = 5; p < 0.004, Two-way ANOVA).

The observed transitions and transversions in mtDNA were analysed, and the percentage of each type is shown in Table 4. G>A/C>T transitions increased by almost2-fold to 27% in patients carrying the *ITPA 94A* allele, compared to 16% in patients homozygous for the *ITPA 94C* wildtype allele. In contrast, a slight decrease in A>G/C>T transitions to 59% from 65% was detected in patients carrying the variant *ITPA*. Moreover, there was a slightly lower decrease in transversions to 14% compared to 19%, among patients carrying the variant *ITPA*.

**Table 4:** Transitions and transversions among homoplasmic and heteroplasmic mtDNA mutations in AHM patients

***ITPA***	**Total**	**Transitions**		**Transversions**
**genotype**	**mutations**	**A>G/T>C**	**G>A/C>T**	
4 homozygous *94C* wildtypes:				
Homoplasmic	38	82%	18%	0
Heteroplasmic	11	9%	9%	82%
Total	49	65%	16%	19%
13 carrier *94A* mutants:				
Homoplasmic	329	66%	32%	2%
Heteroplasmic	92	33%	8%	59%
Total	421	59%	27%	14%

Mutations shown as total numbers and percent of total, in 13 patients carrying the *ITPA 94A* variant allele compared with 4 patients homozygous for the *ITPA 94C* wildtype allele. *ITPA 94A* produced 12 unique point mutations; 7 insertions were also identified.

### N-calls

Failure of the MitoChip v2.0 software to assign a base to any position is termed a no-call or ‘N-call.’ N-calls were analysed using two software methods, GSEQ and sPROFILER (see Methods) for both MitoChip v2.0 sections (i.e., the reference sequence and common haplotypes). Table 5 illustrates that the N-calls were slightly higher for patients carrying the *ITPA 94A* allele compared to those homozygous for the *ITPA 94C* wildtype allele in both MitoChip v2.0 sections, when GSEQ software determined the base calls. A significant reduction in N-calls occurred when further analysed by sPROFILER software (p < 0.0001 in patients carrying the *ITPA 94A* allele, and p< 0.05 in patients were homozygous for the *ITPA 94C* wildtype allele).

**Table 5:** Mean of N-calls in mtDNA of AHM patients

**mtDNA N-calls**	**Patients carrying**			**Patients homozygous for**		
**(MitoChip v.2.0)**	***ITPA 94A*variant allele (n=13)**			***ITPA 94C*wildtype allele (n=4)**		
	**GSEQ**	**sPROFILER**		**GSEQ**	**sPROFILER**	
rCRS	588	264	p<0.0001	532	234	p<0.05
Additional tiling	6406	4147	p<0.0001	6346	4075	p<0.05

mtDNA from 13 patients carrying the *ITPA 94A* variant allele compared with 4 patients homozygous for the *ITPA 94C* wildtype allele. Unique mutations were identified on the MitoChip v.2.0, and are analysed as those identified by the rCRS section of the microarray *vs.* the additional tiling section for common haplotypes. N-calls were analysed using proprietary GSEQ then sPROFILER software; p values calculated using Mann–Whitney test comparing total N-calls obtained from GSEQ and sPROFILER software.

**Figure 5:**
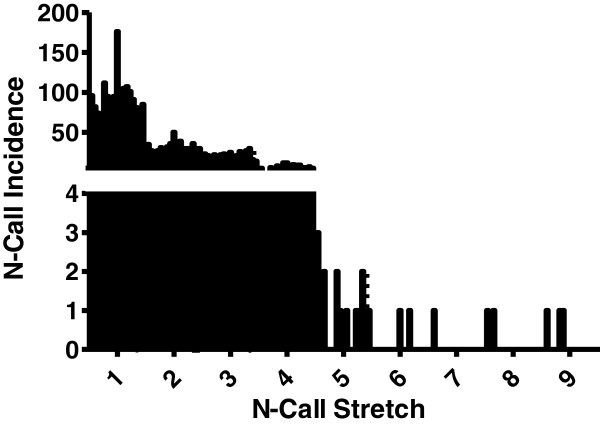
**Incidence of N-calls following sPROFILER analysis of the entire mtDNA genome.** This illustrates the total distribution of continuous stretches of N-calls in mtDNA (rCRS section) in both sets of AHM patients, those carrying the *ITPA 94A* mutation and those homozygous for the *ITPA 94C* wildtype allele. Continuous stretches of >5 N-calls were re-analysed by conventional sequencing.

Further analysis was made of continuous stretches of N-calls after analysis by sPROFILER software. Short N-calls stretches of 2–5 undefined bases were found in AHM patients with and without the *ITPA 94A* allele (Figure 5). However, in each of 7 of the 13 patients carrying the *ITPA 94A* allele we observed at least one longer continuous stretch (6–9 undefined bases) of N-calls, compared to an absence of longer N-call stretches in the patients homozygous for the *ITPA 94C* allele. Thus only patients carrying the *ITPA 94C>A* variant were found to have long continuous stretches of N-calls.

In brief, conventional sequencing of mtDNA samples with long N-call stretches revealed that the 5 MDS patients carrying the *ITPA 94A* variant allele had unique mutations and insertions compared to the reference sequence, while no unique mtDNA mutations were detected in the 2 MDS patients who were homozygous for the *ITPA* wildtype allele. Interestingly, our analysis using Haplogrep software found that there were also 11 haplogroups that were missing 27 expected homoplasmic variants. All but one of these variants was assigned by the MitoChip v.2.0 as an N-call.

### Association of haematological malignancy with ITPA polymorphic sequence variant frequency

From the total of 85 AHM patients (170 alleles), 11 were heterozygous for the *ITPA 94A* mutant exonic allele, 20 were heterozygous for the intronic *ITPA IVS2+21C* mutant allele, 2 were compound heterozygous *94A/IVS2+21C*, and 1 was homozygous *IVS2+21C*. The overall frequency for these patients for the *ITPA 94A* mutant allele (7.7%) was not significantly different from both our published frequency [3] and Marsh *et al.*[13] of 6% for the allele in normal healthy Caucasian populations. For the *ITPA IVS2+21C* mutant allele, the overall AHM frequency (14.1%) was similar to our published value of 13%.

**Figure 6:**
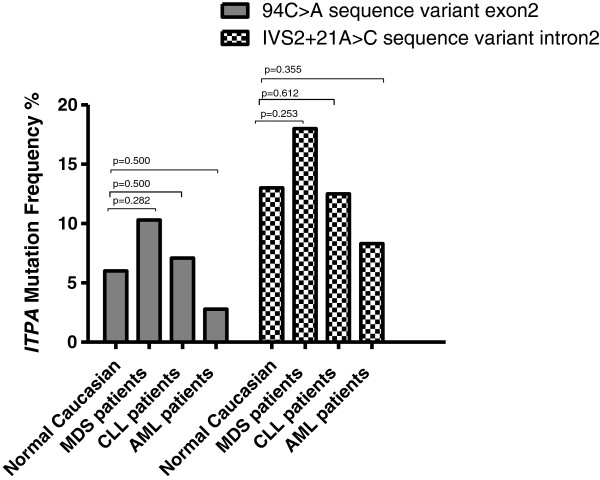
**Frequencies of the *ITPA *polymorphism in normal Caucasian population compared with three AHM groups.** The % frequencies of the *ITPA 94A* and *IVS2+21C* sequence variants are shown. AHM groups examined were MDS (n=39), CLL (n=28), and AML (n=18). The p-values were calculated using Fisher’s exact test. The frequency for the normal population is from Sumi *et al.*[3].

When the AHM group was then broken down into the 3 disease groups and the frequencies calculated separately, the frequencies of the *ITPA 94A* and the *IVS2+21C* alleles were higher for the MDS disease group (10.3% and 18% respectively) than control populations but not significantly (p= 0.282 and 0.253 respectively). For CLL, these frequencies were similar to controls, while for AML the two mutant alleles were less frequent but not significantly. Thus the results were not statistically different for AHM overall, but the *ITPA* sequence variants tended to be more frequent for MDS (Figure 6): a larger study group is required than was available for this pilot study.

## Discussion

Nucleotide imbalance is normally corrected by hydrolytic nucleotidases [4] and phosphorylases [24], which prevent accumulation of harmful non-canonical (‘rogue’) nucleotides and nucleosides and their incorporation into DNA, as well as breaking down and recycling endogenous nucleotides [36]. ITPase is one of 4 highly-conserved groups of nucleotidases that specialize in monitoring rogue nucleotides, the others being the Nudix enzyme superfamily, the deoxy-UTPases, and the α-nucleotide pyrophosphatase superfamily.

‘Genotoxic stress’ has been proposed to be an early event in cell transformation, but the mechanisms for this have remained unknown [27]. One mechanism of genotoxic stress that has been more widely proposed is ROS-catalysed oxidation producing rogue nucleotides in DNA [15,16]. However, it has been shown that accumulation of ITP/dITP can also lead to DNA instability [8]. Reduced ITPase activity produces accumulation of the rogue nucleotides ITP and dITP, as well as xanthosine triphosphate (XTP) [37], and these rogue nucleotides may potentially increase the mutational load in mitochondria, slowly leading towards loss of cellular integrity, with either of two outcomes: cell death (by apoptosis) or malignancy. An *in vitro* study on HeLa cells with a knockdown *ITPA* gene has illuminated the role of ITPase in maintaining DNA integrity and preventing apoptosis in human cells. The study concluded that absence of functional ITPase activity caused by *ITPA* gene defects may lead to DNA damage and cancer as a consequence of the accumulation of non-canonical nucleotides [11]. The incorporation of non-canonical nucleotides arising from drugs can also affect mtDNA. A recent study by Daehn et al. (2011) described the effect of incorporation into human mtDNA of 6-thioguanine, a thiopurine drug concluding that this may explain some adverse thiopurine reactions including hepatotoxicity and myopathy [38], although the study did not specifically examine ITPase effects.

We therefore considered that chronic genotoxic stress particularly may affect mtDNA of bone marrow cells, producing late-onset and pre-cancerous cell transformation which may progress to cellular constitutional imbalance and hematologic malignancy. In particular, a role for *ITPA* polymorphism in the production of mitochondrial DNA mutations has not been previously considered. We maximized the chances of finding mtDNA damage by studying high-turnover cells (from bone marrow) in older adults. In addition, we aimed to assess the incidence of the *ITPA* allelic variants in AHM patients compared to the normal population.

Before mtDNA sequences could be analysed meaningfully for mutations, we assigned haplogroups to each of the patients who were confirmed carriers of the *ITPA 94A* variant. Removing haplogroup (non-pathogenic) variants using HaploGrep [33], based on data from Phylotree [34] revealed unique mutations. The absence of some expected haplogroup variants was intriguing, but we were able to assign these absences to the performance of the MitoChip array, where they were assigned as N-calls, as discussed below. After haplogroup correction, there was a significant increase in the total number of mutations among 10 of the 13 patients carrying the *ITPA 94A* mutant allele, compared with the 4 patients with wildtype *ITPA*. However, when homoplasmic and heteroplasmic mutations were examined separately, only the heteroplasmic showed a significant increase.

Alterations in mtDNA have been identified and associated with solid tumors in bladder, breast, colon, head and neck, kidney, liver, lung and stomach, and in the hematologic malignancies leukemia and lymphoma [39]. One study of 104 MDS patients showed a high mtDNA mutation frequency *in the D-loop,* transfer RNA and ribosomal RNA genes, with low mtDNA mutation frequency in protein encoding genes suggesting an association of onset of MDS with mitochondrial abnormalities [40]. About 40% of CLL and AML and 50% of MDS patients had increased mtDNA somatic point mutations.

The mitochondrial genome comprises 37 discrete genes, of which the short ‘control region’ (or *D-loop*), contains the major elements for mtDNA replication and transcription, and is a mutational hotspot region in some human cancers [41]. Analysis of mitochondrial genes, including by microarray based re-sequencing, may represent a powerful tool for early detection in hematologic malignancies and tumors. For example, research has suggested there is an increased risk of MDS, CLL and several other cancers [26,41,42] when mutations occur in the ‘protein-encoding region’ (or *Prot*) of mtDNA, which contains 13 genes encoding respiratory chain and oxidative phosphorylation (OXPHOS) enzymes. These genes – NADH dehydrogenase (*MT-ND*), cytochrome c oxidase (*MT-CO*)*,* the ATPases (*MT-ATP6*) and cytochrome *b* (*MT-CYB*) – are highly transcribed. This may leave them more susceptible to mutational agents, such as rogue nucleotides. These genes were observed to have a high level of mutations, which may then compromise mitochondrial function and cellular integrity. On the other hand, there is less evidence of the involvement of the RNA-encoding regions, containing the genes for 2 ribosomal RNA genes (*rRNA*) and 22 transfer RNA genes (*tRNA*).

Interestingly, our analysis showed that AHM patients carrying the *ITPA* sequence variant had increased rates of both homoplasmic and heteroplasmic mutations in the *rRNA*, *tRNA*, and protein encoding regions where *ITPA* wildtypes had no mutations, compared with mutations frequencies up to 1.6% in *ITPA* mutant carriers. The *Prot* and OXPHOS regions also showed increases (see Table 3 and Figure 4).

Overall, there was a significant increase in total mutation rates in mtDNA of patients carrying the *ITPA 94A* mutation. This implies that the functionality of critical mitochondrial genes can be compromised by ITP/dITP accumulation because the high transcriptional activity of these genes may make them more susceptible to mutational agents such as rogue nucleotides. The role of ITPase in cellular surveillance of rogue nucleotides may thus have late-onset effects in humans, with *ITPA* gene defects having consequences for genomic stability.

The case of ITPase-deficient mice has provided direct *in vivo* evidence that deoxy-ITP (dITP) can be incorporated at high levels into DNA, resulting in death before weaning [8]. Furthermore, Spee *et al.*[43] demonstrated *in vitro* that when dITP was added to dNTPs in PCR amplification of the 171 bp *Lactococcus lastis nisZ* gene, numerous point mutations were generated. In addition, the type of mutation generated occurring appears to be consistent: for *Lactococcus*, the major point mutations generated by dITP were transitions (60% A>G/T>C and 26% G>A/C>T), with only 14% transversions and low frequencies of insertions or deletions. Kamiya *et al.*[44], using transfection of mutant *ITPA* into NIH-3T3 cells, showed that deoxy-xanthosine exclusively induces G>A transitions in mammalian cells by introducing deoxy-xanthosine into a synthetic gene. This was confirmed again by Kamiya *et al.*[45] when they utilized DNA synthesis experiments with synthetic templates containing xanthine and hypoxanthine - which form the rogue nucleotides dXTP and dITP - to show that G>A and A>G transitions were generated, demonstrating that these nucleotides can be highly mutagenic [46].

These results match well with our findings (Table 4). There was a 2-fold increase in the percentages of total G>A/C>T transitions accompanied by a slight percent decrease of A>G/T>C transitions in mtDNA of patients carrying the *ITPA 94A* mutant allele, compared to patients who were wildtype for *ITPA*. Transversions were slightly decreased. This suggested mutational selectivity in mtDNA transitions caused by low activity (<25% normal) of ITPase.

Transversions are base changes resulting from purine-pyrimidine substitutions or vice versa, in theory caused by misincorporation following oxidation/deamination of DNA bases by ROS. The rise in G>A/C>T transitions were caused in theory by misincorporation of rogue nucleotides, such as dITP. However, the detection of any expected insertions or deletions (‘indels’) arising from the presence of the *ITPA 94A* polymorphism was initially limited by the nature of the MitoChip v.2.0 and GSEQ software, which is not designed to detect indels. Yet deletions were of particular interest, given that deletion/depletion is common in other mitochondrial metabolism disorders such as thymidine phosphorylase (MNGIE), thymidine kinase (TK2) and deoxy-guanosine kinase (dGK) deficiencies [25,47].

The MitoChip’s GSEQ software has no provision for detecting either insertions or deletions, assigning them as ‘N-calls.’ We predicted that indels may be assigned as ‘N-calls’ by the GSEQ software, and in particular that the frameshift produced by an indel may result in a length of adjacent N-calls. Therefore, continuous stretches of N-calls in the MitoChip reference sequence section were examined in depth, using a second base-assignment software sPROFILER, which was designed to reduce the number of N-calls [45].

When we used sPROFILER software, it produced a significant drop in N-calls in both patient sets (Table 5). In particular, we observed continuous stretches of N-calls - ranging from 6 to 9 bases – only among AHM patients carrying the *ITPA 94A* mutant allele (Figure 5). Thus by using sPROFILER we managed to narrow the search area for any sequence variants or indels to be then analysed by conventional sequencing. By focusing on the longest stretches of N-calls in each sample we were able to identify unique sequence variances and insertions [48]. Deletions were not found, which points to a mutational mechanism for low ITPase that varies from that of, of example, MNGIE. This finding supported our hypothesis that the presence of the *ITPA 94C>A* sequence variant may lead to an increase in mtDNA mutations (specifically transitions), which will subsequently cause insertions/deletions, particularly among MDS patients.

Finally, we considered whether ITPA deficiency might be causative of some forms of adult (i.e. late-onset) haematological malignancy (AHM). We found that the frequencies of *ITPA* variant alleles for the 3 (Caucasian) AHM groups examined were raised for MDS and CLL – but not statistically different – when compared to the normal Caucasian population. For AML – one of the mixed-lineage leukemias – *ITPA* variants were at a lower frequency than normal (Figure 6). In retrospect, our study would have been underpowered to detect a significant contribution of reduced ITPase activity to the pathogenesis of AHM, especially as *ITPA* polymorphism may be only one of several causative factors for a rogue nucleotide-driven DNA repair pathology that predispose to DNA instability. However, we suggest that the observed trends warrant screening of larger numbers of MDS patients in particular.

The reduced frequency of mutant *ITPA* that we observed for AML may point towards a similar mechanism as reported for MLL, but this requires further study. *ITPA* is one of 5 genes noted to be up-regulated in adult mixed-lineage leukemias [10], which is associated with therapy-related AML. Treatment for CLL with the purine (rogue) analogue fludarabine in combination chemotherapy achieves high response rates, but has also been associated with treatment related MDS and AML in up to 10% [49]. It is therefore possible that ITPA may be involved in genetic instability leading to these secondary marrow disorders, although we could not make any firm conclusions from our present study.

## Conclusion

Significant changes in the levels of total mtDNA mutations found when the *ITPA 94A* mutant allele was present; indicate that reduced ITPase activity in humans may not be as benign as previously considered. The nature of these mtDNA mutations, i.e. predominantly G>A/C>T transitions, were consistent with our hypothesis that these were induced by deoxy-ITP/XTP rogue nucleotides. Insertions were also found among some of the MDS patients. The role of ‘house-cleaning’ genes in cell transformation remains to be investigated further, but our small study presents evidence towards a new paradigm to explain the early stages of cellular transformation leading to adult malignant disease.

## Competing interests

The authors have no conflict of interest with any company or financial organization.

## Authors’ contributions

MZ the primary author, designed and conducted the study; JD co-designed the study and co-wrote the paper; GP co-designed the study and co-wrote the paper; DV co-heads the Mater Pathology OMICS laboratory, provided continuing logistical support and reviewed the paper; RWT and JWY provided the conventional mtDNA sequencing and interpretation of mtDNA haplogroups and contributed to writing; LC is a hematologist, participating in sample provision, analysed patient clinical data and reviewed and edited the paper; TF co-supervised the project and reviewed the paper; AM provided the initial concept for the study and reviewed the paper; and FB was study chair, co-designed the study, and reviewed the paper. JD and FB are both designated as senior authors. Authors had access to the primary clinical data. All authors read and approved the final manuscript.

## Supplementary Material

Additional file 1: Table S1 Homoplasmic mtDNA mutations identified. This is including the haplogroups analysis of mtDNA from 13 adult haematological malignancies (AHM) patients carrying the *ITPA 94C>A* sequence variant. **Table S2.** Heteroplasmic mtDNA mutations identified. This is including the haplogroups analysis of mtDNA from 13 adult haematological malignancies (AHM) patients carrying the *ITPA 94C>A* sequence variant. **Table S3.** Homoplasmic and heteroplasmic mutations identified. This is including the haplogroups analysis of mtDNA from 4 adult haematological malignancies (AHM) patients carrying the wildtype *ITPA* (controls). Click here for file
