# Supplementary material for: Inosine Triphosphate Pyrophosphohydrolase (ITPA) polymorphic sequence variants in adult hematological malignancy patients and possible association with mitochondrial DNA defects
Source: J Hematol Oncol. 2013 Mar 29;6:24. doi: 10.1186/1756-8722-6-24 (PMC3765497; doi:10.1186/1756-8722-6-24)
Supplement: Additional file 1: Table S1 — Homoplasmic mtDNA mutations identified. This is including the haplogroups analysis of mtDNA from 13 adult haematological malignancies (AHM) patients carrying the ITPA 94C>A sequence variant. Table S2. Heteroplasmic mtDNA mutations identified. This is including the haplogroups analysis of mtDNA from 13 adult haematological malignancies (AHM) patients carrying the ITPA 94C>A sequence variant. Table S3. Homoplasmic and heteroplasmic mutations identified. This is including the haplogroups analysis of mtDNA from 4 adult haematological malignancies (AHM) patients carrying the wildtype ITPA (controls). [file 1756-8722-6-24-S1.pdf]

**Table S1. Homoplasmic mtDNA mutations identified. This is including the haplogroups analysis of mtDNA from 13 adult haematological malignancies (AHM) patients carrying the *ITPA 94C>A* sequence variant.**

| **Sample ID** | AML_6 | CLL_12A | CLL_20 | CLL_21 | CLL_4 | MDS_11A | MDS_13 | MDS_6 | MDS_6A | MDS_9 | MDS_20 | MDS_21 | MDS_22 |
|---|---|---|---|---|---|---|---|---|---|---|---|---|---|
| **Haplogroup** | H4a1a1 | T2a | K1b1a1 | U3a1 | U5a2a4 | H3b | K1 | U5a2 | H3g | U5a1a1 | K1a4a1b1 | N1a | U5a2c |
| **Homoplasmic Variants** | 73G | 73G | 73G | 73G | 73G | 153G | 73G | 73G | 152C | 73G | 73G | 73G | 73G |
| | 263G | 263G | 152C | 150T | 263G | 263G | 263G | 200G | 263G | 263G | 263G | 199C | 263G |
| | 750G | 709A | 199C | 263G | 750G | 750G | 750G | 263G | 750G | 750G | 750G | 204C | 750G |
| | 1438G | 750G | 263G | 750G | 1438G | 1438G | 1189C | 750G | 1438G | 1438G | 1189C | 263G | 1438G |
| | 3992T | 1438G | 750G | 1438G | 2706G | 2581G | 1438G | 1438G | 3144G | 1700C | 1438G | 669C | 2706G |
| | 4024G | 1888A | 1189C | 1811G | 4232C | 4769G | 1811G | 2706G | 4769G | 2706G | 1811G | 750G | 3421A |
| | 4769G | 2706G | 1438G | 2294G | 4655A | 5147A | 2706G | 4769G | 6776C | 4769G | 2706G | 1438G | 4769G |
| | 5004C | 2850C | 1811G | 2706G | 4769G | 6776C | 3483A | 7028T | 8860G | 5495C | 3480G | 1719A | 7028T |
| | 5276G | 4216C | 2706G | 3010A | 7028T | 8860G | 4769G | 7843G | 10754C | 7028T | 4769G | 2702A | 8860G |
| | 8269A | 4769G | 3480G | 4703C | 8860G | 13813A | 5581G | 8676T | 12236A | 8860G | 6260A | 2706G | 10619T |
| | 8860G | 4917G | 4646C | 4769G | 9477A | 15326G | 7028T | 8860G | 13611G | 9477A | 7028T | 2758A | 11467G |
| | 9123A | 4931T | 4769G | 6518T | 11467G | 16111T | 8860G | 9389G | 15326G | 11467G | 8098G | 4769G | 11719A |
| | 10044G | 7028T | 5913A | 7028T | 11719A | 16129A | 9055A | 9477A | 15496G | 11719A | 8856C | 5315G | 12308G |
| | 14582G | 7740G | 7028T | 8860G | 11893G | | 9554A | 11204C | 16325C | 12308G | 8860G | 7028T | 12372A |
| | 15326G | 8697A | 7364G | 9266A | 12308G | | 9698C | 11467G | 16519C | 12372A | 9055A | 8860G | 13617C |
| | | 8860G | 8860G | 10506G | 12372A | | 10398G | 11719A | | 14766T | 9698C | 8901G | 14766T |
| | | 11251G | 9055A | 11467G | 13827G | | 10410C | 12308G | | 14793G | 10398G | 10238C | 14793G |
| | | 11719A | 9698C | 11719A | 13928C | | 10550G | 12372A | | 15218G | 10550G | 10398G | 15326G |
| | | 11812G | 9962A | 12308G | 14766T | | 11299C | 13474C | | 15326G | 11299C | 10688A | 16270T |
| | | 13368A | 10289G | 12372A | 14793G | | 11467G | 14587G | | 15924G | 11467G | 11719A | 16526A |
| | | 13965C | 10398G | 13934T | 15326G | | 11719A | 14766T | | 16256T | 11485C | 12501A | |

| | | | | | | | | | | | | | |
|---|---|---|---|---|---|---|---|---|---|---|---|---|---|
| | | 14233G | 10550G | 14139G | 16114A | | 11914A | 14793G | | 16270T | 11719A | 12705T | |
| | | 14766T | 11299C | 14766T | 16270T | | 12308G | 15326G | | 16399G | 11840T | 13780G | |
| | | 15119A | 11467G | 15326G | 16294T | | 12372A | 15758G | | | 12308G | 14766T | |
| | | 15326G | 11719A | 16390A | 16526A | | 14167T | 16104T | | | 12372A | 15043A | |
| | | 15452A | 11923G | 16519C | | | 14766T | 16256T | | | 13740C | 15317A | |
| | | 15607G | 12308G | | | | 14798C | 16270T | | | 14167T | 15326G | |
| | | 15928A | 12372A | | | | 15326G | 16526A | | | 14766T | 15833T | |
| | | 16126C | 13967T | | | | 16311C | | | | 14798C | 16147G | |
| | | 16294T | 14167T | | | | 16519C | | | | 15326G | 16172C | |
| | | | 14766T | | | | | | | | 16311C | 16223T | |
| | | | 14798C | | | | | | | | 16519C | 16248T | |
| | | | 15257A | | | | | | | | | 16300G | |
| | | | 15326G | | | | | | | | | 16519C | |
| | | | 16311C | | | | | | | | | | |
| | | | 16319A | | | | | | | | | | |
| | | | 16463G | | | | | | | | | | |
| | | | 16519C | | | | | | | | | | |

**Table S2. Heteroplasmic mtDNA mutations identified. This is including the haplogroups analysis of mtDNA from 13 adult haematological malignancies (AHM) patients carrying the *ITPA 94C>A* sequence variant.**

| **Sample ID** | AML_6 | CLL_12A | CLL_20 | CLL_21 | CLL_4 | MDS_11A | MDS_13 | MDS_6 | MDS_6A | MDS_9 | MDS_20 | MDS_21 | MDS_22 |
|---|---|---|---|---|---|---|---|---|---|---|---|---|---|
| **Haplogroup** | H4a1a1 | T2a | K1b1a1 | U3a1 | U5a2a4 | H3b | K1 | U5a2 | H3g | U5a1a1 | K1a4a1b1 | N1a | U5a2c |
| **Heteroplasmic Variants** | 5002C | 708A | 916T | 1812G | 11466G | 13037A | 70T | 198A | 13037A | 2721A | 6967A | 671C | 9473G |
| | 8076A | 4772A | 1191A | 2298C | 12307G | 14118G | 1815G | 566A | 16326C | 11466G | 8858C | 13037A | 11466G |
| | 8898A | 10464C | 1193C | 3009A | 12309G | | 5580G | 8678C | | 12307G | 8898A | 16250T | 12307G |
| | 13037A | 13037A | 7365G | 11466G | 13619C | | 9054A | 11202C | | 12309G | 11466G | 16305G | 12309G |
| | | 14765T | 9054A | 12307G | 14765T | | 9555A | 11210C | | 13037A | 12307G | 16355T | 13619C |
| | | 15327G | 11466G | 12309G | 16196A | | 11466G | 11466G | | 13619C | 12309G | | |
| | | | 12307G | 13037A | | | 11908T | 12309G | | 14765T | 13037A | | |
| | | | 12309G | 15844G | | | 12307G | 13037A | | | | | |
| | | | 13037A | | | | 12309G | 13473A | | | | | |
| | | | 14165T | | | | 13037A | 13619C | | | | | |
| | | | 14169T | | | | 14765T | 14591A | | | | | |
| | | | 15327G | | | | | 14771A | | | | | |
| | | | 15378G | | | | | 14855T | | | | | |
| | | | 15948C | | | | | 15327G | | | | | |
| | | | | | | | | 15757G | | | | | |

**Table S3. Homoplasmic and heteroplasmic mutations identified. This is including the haplogroups analysis of mtDNA from 4 adult haematological malignancies (AHM) patients carrying the wildtype *ITPA* (controls).**

| Sample ID | AML_1 | CLL_8 | MDS_16 | MDS_18A |
|---|---|---|---|---|
| Haplogroup | H5 | H2a2 | H5 | H3b |
| Homoplasmic Variants | 263G | 263G | 263G | 153G |
| | 456T | 3882A | 456T | 263G |
| | 750G | 5460A | 750G | 750G |
| | 1438G | 8860G | 1438G | 1438G |
| | 4769G | 15326G | 4769G | 2581G |
| | 8860G | 16235G | 8860G | 4769G |
| | 15326G | | 9083C | 5147A |
| | 16304C | | 15326G | 6776C |
| | | | 16304C | 8860G |
| | | | 16311C | 13813A |
| | | | 16519C | 15326G |
| | | | | 16111T |
| | | | | 16519C |
| Homoplasmic Variants | 4771T | 5459A | 4771G | 4772A |
| | 9987C | 13037A | 16312C | 13037A |
| | 13037A | 15327G | | |
| | | 16296G | | |
